# Simulation of directional crack propagation by energy-absorbing blasting in deep gob-side entry retaining under different lateral pressure coefficients

**DOI:** 10.1371/journal.pone.0336444

**Published:** 2026-02-09

**Authors:** Nan Liu, Chuanqing Guo, Sudong Bao, Tao Wang, Feng Zhang, Zhongcheng Qin

**Affiliations:** 1 School of Energy and Mining Engineering, Shandong University of Science and Technology, Qingdao, China; 2 Jining No. 2 Coal Mine, Yankuang Energy Group Company Limited, Jining, China; University of Sharjah, UNITED ARAB EMIRATES

## Abstract

To address the challenges associated with directional roof-cutting through energy-absorbing blasting under high-stress conditions in deep gob-side entry retaining, this study combines numerical simulations and field tests to investigate the effects of varying lateral pressure coefficients (k) on crack propagation. Numerical simulations, utilizing an HJC constitutive model and an element erosion criterion, demonstrate that: (1) a higher lateral pressure coefficient significantly inhibits crack propagation, resulting in a reduction of main crack length and a simplification of crack morphology, while simultaneously increasing the proportion of elastic vibration energy; (2) optimal crack control is achieved when the energy-absorbing direction aligns with the maximum in-situ principal stress direction. The on-site verification at the 13303 working face demonstrates that aligning the opening of the energy-concentrating pipe with the direction of the roadway, which corresponds to the direction of the maximum principal stress, results in a directional main fracture occurrence rate exceeding 95%. significantly enhancing the retained roadway’s quality. These findings provide a theoretical foundation for precise blasting design in deep high-stress mining environments.

## 1. Introduction

In deep coal mining, the adoption of gob-side entry retaining technology serves as an effective method to enhance resource recovery rates and mitigate the conflict between extraction and development. [1–2]. The success of this technique hinges on the formation of directional fractures through pre-split blasting for roof cutting, a process whose effectiveness is critical to the stability of the retained entry. [3]. To achieve precise control over roof fracturing, shaped charge blasting has been introduced and widely implemented, leveraging its unique capability to concentrate and direct energy release. [4–5].

Extensive research has been conducted into the underlying mechanisms, such as jet-induced fracturing [6–7], as well as key operational parameters including borehole spacing [8–9] and permeability enhancement effects [10–11]. These studies have yielded substantial insights and established a solid theoretical foundation for the technology.

However, when shaped charge blasting is applied in deep high-stress environments, the reliability and adaptability of its roof-cutting effect face severe challenges [12]. Deep rock masses exist within a coupled field of high in-situ stress and blasting dynamic loads, leading to fundamental changes in their fracture mechanisms [13–14]. Field practice indicates that neglecting the dominant role of in-situ stress often results in insufficient fracture propagation, directional deviation, or even failure of pre-splitting [15]. Scholars at this stage have pointed out that the in-situ stress field, particularly the lateral pressure coefficient, is a key variable controlling blasting damage and crack propagation [16–17]. Existing research confirms the inhibitory or guiding effect of in-situ stress on cracks, but it predominantly focuses on phenomenological description and qualitative analysis.

The root of the problem lies in the fact that deep rock masses are situated in a high in-situ stress environment, where the magnitude of the initial stress field often far exceeds that of blasting dynamic stresses, thereby dominating the final propagation behavior of cracks. Traditional shaped charge blasting designs based on shallow conditions fail to adequately account for this decisive factor. Although studies have recognized the core influence of in-situ stress (especially the lateral pressure coefficient k, which characterizes the difference in horizontal stresses) [18–19] and have observed its inhibitory [20] or guiding effects on cracks [21], a quantitative and mechanistic understanding is still lacking regarding how the lateral pressure coefficient dynamically affects and regulates the entire process of shaped charge blasting crack initiation, propagation, and arrest under deep high-stress conditions. There is a failure to systematically reveal the quantitative influence mechanism of key in-situ stress parameters (such as the lateral pressure coefficient k) on shaped charge blasting energy propagation and crack extension during dynamic changes. This creates a gap between current understanding and the complex, variable real geomechanical environment in deep strata. Consequently, a discrepancy exists from theoretical cognition to on-site process optimization, hindering precise blasting design tailored to specific in-situ stress conditions.

Therefore, based on the engineering context of deep gob-side entry retaining in the Jining Mining Area of Shandong Province, this paper aims to combine numerical simulation and field testing to quantitatively analyze the synergistic patterns of blasting energy field distribution, crack propagation paths, damage evolution, and dynamic pressure relief effects under different lateral pressure coefficients (k). The research outcomes seek to reveal the crack control laws of shaped charge blasting in high-stress environments, apply the derived laws to field trials, and ultimately achieve the goal of improving the formation quality and control effectiveness of gob-side entry retaining. This will provide a reference for the safe and efficient extraction of deep resources.

## 2. Engineering background and site investigation

### 2.1. Geological conditions

**Fig 1:**
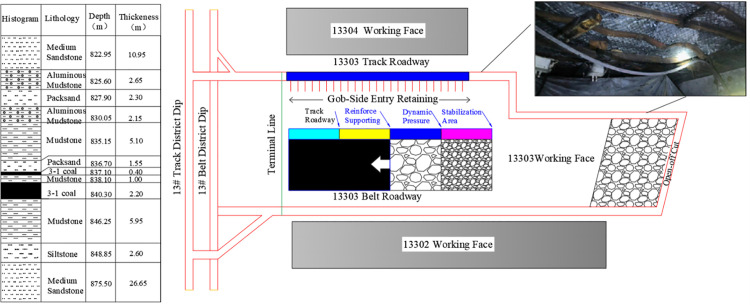
Layout and Roof Conditions of the 13303 Working Face in a Certain Mine.

The mining depth of a coal mine in Jining, Shandong Province, has surpassed 800 m. The primary mining zones are the 3_upper_ and 3_lower_ coal seams, with the first mining face of the 13th area centrally located. The average roadway elevation ranges from −765. to −799.8 m. Surrounding rock exhibits low strength and a developed fracture structure. The geological conditions of the 13303 working face are complex and variable, featuring a local mudstone pseudo-roof on the coal seam. This rock type is characterized by low strength, high plasticity, and fragility, posing a risk of caving. The immediate roof consists of fine sandstone, which is harder than the pseudo-roof but still prone to local fractures. Above this, thick-layered mudstone is soft and susceptible to separation, leading to roof instability. The coal seam floor is composed of mudstone with significant thickness variation, which softens upon water exposure, greatly reducing its bearing capacity. Fig 1 illustrates the working face layout and roof conditions.

### 2.2. Working face condition

The working face, situated centrally in the 13th mining area, is the area’s inaugural mining face. It spans 140–220 meters in length, with an advancement length of 1201.58 m. A geophysical fault, 13F^13^, with a height ranging from 0 to 30 meters, lies to the south, necessitating a knife-shaped configuration that expands as mining progresses.

Currently, urban planning and development constraints significantly hinder access to high-quality coal resources, leading to a marked decline in effectively recoverable reserves and exacerbating mining tensions. Additionally, deep mining operations encounter “three high” conditions—high stress, geothermal temperature, and karst water pressure—substantially elevating safety risks. Ground stress measurements at the 13303 working face reveal that the maximum principal stress is nearly horizontal, 1.48 times the vertical stress, with moderate horizontal stress concentration. The azimuth of this maximum stress is 97° in Table 1.

**Table 1:** Measured stress results of primary rock.

principal stress	actual measurement/MPa	dip/°	azimuth/°
σ_1_	26.99	7°	97°
σ_2_	12.49	61°	35°
σ_3_	9.75	28°	176°
σ_v_	18.20		

### 2.3. Analysis of blasting parameters and failure issues

**Fig 2:**
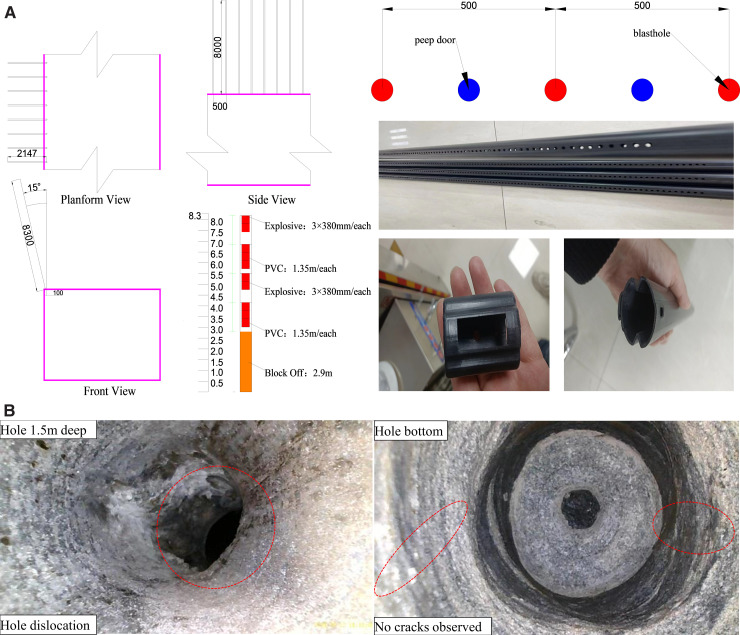
Charge Configuration Diagram (a) Schematic diagram of hole parameters and cartridge form (b) Initial blast hole observation.

The original design for pre-splitting and slotting operations near the 13303 working face involved bidirectional cumulative blasting, guided by the borehole columnar diagram and actual mining conditions. Key parameters included a slotting hole height and depth of 8 m and 8.3 m, respectively, with an angle of 15° (constructed at an upward angle of 75°), borehole diameter of 42 mm, and spacing of 500 mm. Each blasting hole was furnished with four cumulative tubes (38 mm in diameter, 135 mm in length). A 3 + 2 + 3 + 2 explosive charging configuration was implemented, as illustrated in Fig 2a. The chosen explosive was a permissible coal mine explosive, with each cartridge measuring 27 mm in diameter, 380 mm in length, and weighing.24 kg. Digital electronic detonators approved for coal mines were utilized, featuring a decoupled charge structure with a decoupling coefficient of 1.56. The total charge length was 5.4 m, with 2.9 m sealed using a packer bag. The initiation process involved connecting the leg wires in series at the borehole collar.

However, as depicted in Fig 2(b), repeated blasting attempts in the initial trial section of the gob-side entry retaining did not yield the intended outcomes. Field investigations uncovered that due to the combined effects of high in-situ stress and advanced abutment pressure, the pre-splitting boreholes suffered extensive damage, including notable deformation, collapse, shear dislocation, and blockage. These issues hindered charging, reduced energy transfer efficiency, and consequently led to various post-blasting complications such as fragmented fracture seams, inadequate roof-cutting height, and irregular contour surfaces. These shortcomings compromised the entry quality and subsequent support system stability, significantly hindering blasting progress.

The engineering failures underscore two critical scientific challenges necessitating immediate attention: (1) understanding the damage and failure mechanisms of the rock mass surrounding boreholes under high in-situ stress conditions, and (2) elucidating the propagation behavior of cracks resulting from cumulative blasting under the combined influence of static and dynamic loads. Resolving these issues is essential for enhancing blasting design efficiency and ensuring the effective execution of gob-side entry retaining at the 13303 working face.

## 3. Establishment and verification of numerical model for rock mass energy-enhanced blasting

### 3.1. Model establishment

This research focuses on deep rock blasting engineering and utilizes ANSYS/LS-DYNA software for numerical simulation. The Solid164 solid element was chosen to streamline model computation. The model dimensions are 300 cm × 300 cm, with a borehole diameter of 5 cm housing a 3.2 cm explosive cartridge. This setup forms a decoupled charge structure with a decoupling coefficient of 1.56, utilizing air as the coupling medium. The cumulative tube, with a thickness of 0.2 cm, features openings on both ends to enhance directional energy concentration.

This study reduces the three-dimensional blasting problem to a plane-strain model for numerical simulation. This simplification rests on two main considerations. First, the blast hole’s depth is much greater than its diameter, so size effects along the hole axis are significant; the central portion of the hole therefore satisfies plane-strain conditions. Second, the study examines how different lateral pressure coefficients (k) affect crack orientation and length within the profile, rather than attempting to reproduce the full three-dimensional transient process of energy-concentrating jet formation and penetration. A plane-strain model can effectively capture the coupling between the explosion stress wave and the static ground-stress field, as well as the key mechanisms of crack initiation and directional propagation that result. Consequently, the simulation analyzes a single representative section perpendicular to the borehole axis, assigns a normal thickness of one unit (Z = 1 cm), and applies appropriate displacement constraints to that section, thereby reducing the problem to a quasi–two-dimensional model.

**Fig 3:**
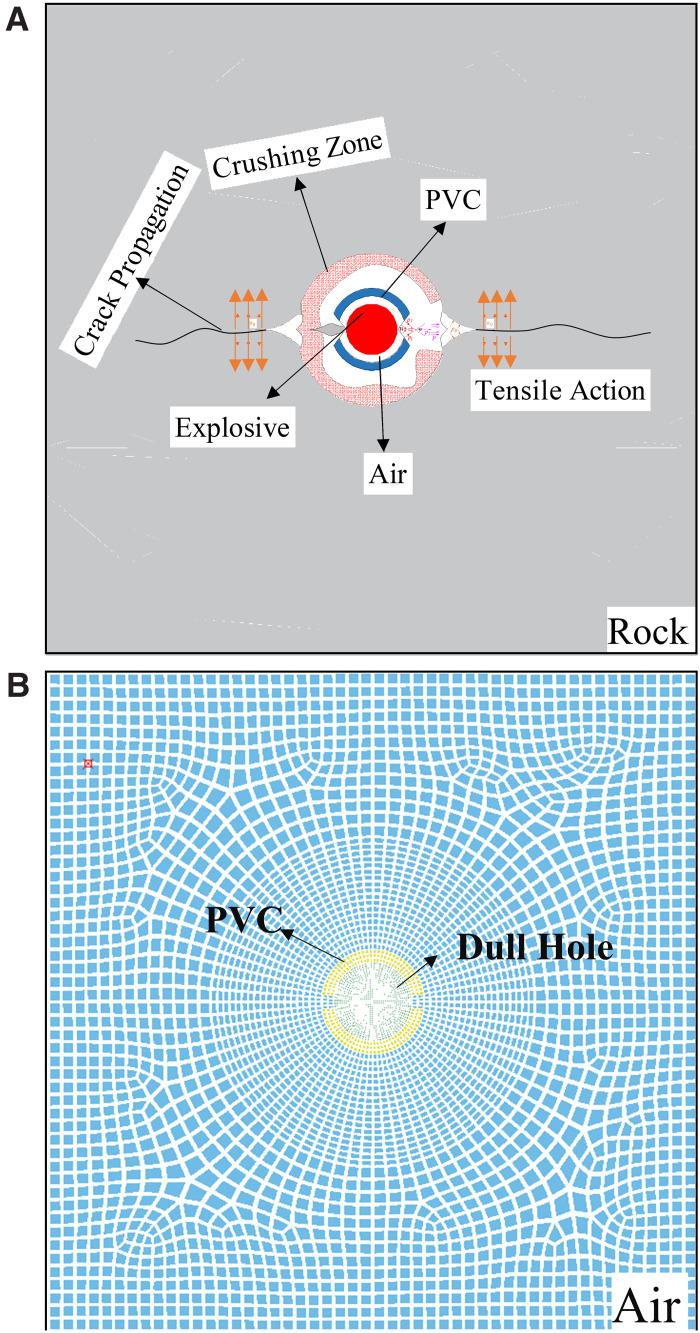
Numerical Model of Shaped Charge Blasting in Rock Masses (a) Schematic Diagram of Shaped Charge Blasting (b) Mesh Division for Hole and Air.

To capture the high-precision dynamics of explosive energy release and to obtain a more accurate description of crack propagation in deep rock masses, the explosive mesh was refined to roughly 1/20 of the rock size [21] The model used the cm-g-μs unit system, and non-reflective boundary conditions were applied along the model edges to approximate an infinite medium. To minimize interference from reflected waves and to account for columnar charging during confining loading and blasting, the time step was set to 10 μs and the total simulation time to 1000 μs. The resulting numerical model employing the fluid–structure coupling algorithm is shown in Fig 3.

### 3.2. Material constitutive behavior and parameter selection

The HJC constitutive model is selected for the rock mass material. However, the standard HJC constitutive model has limitations in simulating the tensile damage of rocks. To more accurately simulate the tensile expansion behavior of explosion-induced cracks, this study defines the tensile and shear failure criteria of elements by adding the keyword *MAT_ADD_EROSION [22]. When the tensile stress reaches 12.1 MPa (the tensile strength of the rock) or the shear strain exceeds 0.01, the rock elements will be damaged and continuously deleted, thus forming the crack expansion. The explosive material model uses the No. 8 constitutive model *MAT_HIGH_EXPLOSION_BURN and the JWL equation of state. The air material adopts the No. 9 material keyword *MAT_NULL and the *EOS_LINEAR_POLYNMIAL equation of state.

The failure simulation strategy used in this study combines two complementary mechanisms. The first mechanism is the damage variable D embedded in the HJC model, which describes progressive cumulative damage and strength deterioration of the material under high pressure and high strain rate. The second mechanism is the additionally introduced erosion criterion (*MAT_ADD_EROSION), which serves as an independent, transient failure criterion. In the LS-DYNA solution framework, these two criteria are evaluated in parallel at each time step.

The *MAT_PLASTIC_KINEMATIC model is chosen for the energy-concentrating tube material. This model disregards plastic deformation and damage accumulation, making it suitable for simulating the initial behavior of the shaped pipe during an explosion without evident failure. Specific model parameters [23] in Tables 2–5.

**Table 2:** Explosive materials and parameters of the equation of state.

Density/(kg/m^3^)	A /GPa	B /GPa	R_1_	R_2_	ω	E_0_ /GPa	V	D /(m/s)	P_CJ_ /GPa
1178	276	8.87	5.2	1.9	0.5	3.82	1.0	3400	9.51

**Table 3:** Air material properties and equation of state parameters.

Density/(kg/m^3^)	C_0_	C_1_	C_2_	C_3_	C_4_	C_5_	V_0_	E_0_
1290	0	0	0	0	0.4	0.4	1	2.5

**Table 4:** HJC rock material parameters.

Limit surface parameters		Damage parameters		Pressure parameters		Mechanical parameters		Efficiency parameters	
A	0.283	D_1_	0.04	p_c_	5.05	ρ/(kg/m^3^)	2610	C	0.003
B	2.537	D_2_	1.0	U_c_	0.0021	fc/MPa	129	–	
N	0.856	EF_min_	0.01	K_1_	0.12	G/GPa	28.72		
Sf_max_	5.05	–		K_2_	0.25	T/MPa	12		
–				K_3_	0.42	E/GPa	32.3		
				P_l_	0.012	υ	0.3		
				Ul	0.012	–			

**Table 5:** Shaped charge liner material properties.

Density/(kg/m^3^)	Modulus of elasticity/GPa	Poisson ratio	Yield stress/MPa	Tangent modulus/GPa	Hardening parameter	Coefficient 1	Coefficient 2
1300	3	0.25	22	0	0	252	5.96

### 3.3. Stress loading scheme

**Fig 4:**
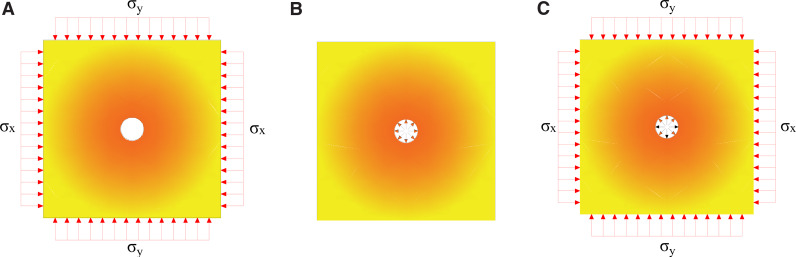
Stress Application Diagram (a) Static load effect (b) Dynamic load effect (c) Superposition effect.

Underground rock blasting occurs within specific stress environments, with varying degrees of impact on crack formation. To investigate how complex stress environments affect crack propagation in deep rock masses, this study examines six different original rock stress field conditions, based on stress measurements from the 13303 working face. In this model stress is applied by Dynain file method [24], the horizontal stress, *σ*_*x*_, the vertical stress, *σ*_*y*_, and the impact load on the hole wall, *Q*_*t*_, are detailed in Fig 4 and Table 6.

**Table 6:** Stress application scheme.

Condition	σ_x_/MPa	σ_y_/MPa	Lateral pressure coefficient
1	0	0	
2	10	10	1
3	9	18	0.5
4	18	18	1
5	27	18	1.5
6	36	18	2

### 3.4. Numerical model validation

**Fig 5:**
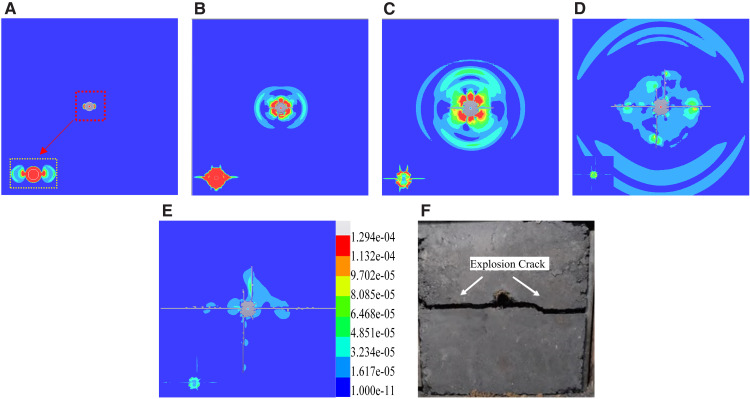
Diagram of Single-Hole Shaped Charge Blasting and Slot Formation Process (a) 8μs (b) 30μs (c) 60μs (d) 120μs (e)300μs (f) Physical Test Results [25].

After establishing the numerical model parameters, we simulated the crack propagation pattern resulting from single-hole cumulative blasting under Condition 1 (0−0MPa), characterized by an absence of initial in-situ stress. Fig 5 depicts the outcome of fracture formation from this simulation. The stress state of the air units is detailed in the magnified view in the bottom-left corner.

The stress waves generated by blasting predominantly traveled in the cumulative direction, leading to preferential extension of main cracks in the same orientation. Cumulative blasting serves to focus explosive energy release along a specific path, effectively inhibiting energy dispersion in other directions and ensuring crack propagation aligns with the intended orientation. To verify the accuracy of the established numerical model and its parameters, simulation results under no in-situ stress conditions (Condition 1) were compared with experimental data from Yang Shuai [25] (Fig 5). The physical experiment illustrated a distinct dominant guided fracture resulting from cumulative blasting, as depicted in Fig 5f. A comparison of the simulation process shown in Figs 5a-e demonstrates that the numerical model accurately reproduced the key observation: blast-induced cracks propagate preferentially along the cumulative direction while suppressing crack development in other directions. The morphology, propagation pattern of the primary crack, and final fracture pattern obtained from the simulation closely matched the experimental results, indicating the model’s ability to effectively capture the directional fracturing mechanism of cumulative blasting. This underscores the reliability of the numerical model and its parameters in studying crack propagation behaviors. Consequently, this validated model can be applied to future simulation studies under varying in-situ stress conditions.

## 4. Simulation results and analysis of blasting damage effects in high-stress rock masses

### 4.1. The stress distribution characteristics of the hole wall under static load

**Fig 6:**
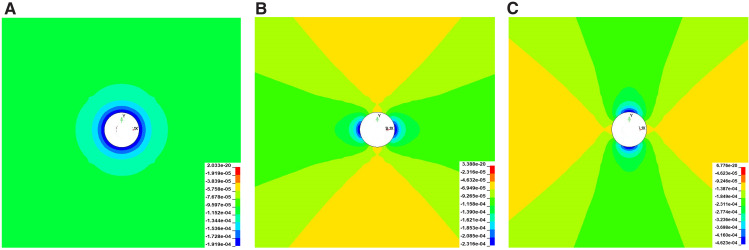
Circumferential Stress Distribution Around the Blasthole (a) *σ*_*x*_ = *σ*_*y*_ = 18MPa (b) *σ*_*x*_ = 9Mpa; *σ*_*y*_ = 18MPa (c) *σ*_*x*_ = 36MPa; *σ*_*y*_ = 18MPa.

**Fig 7:**
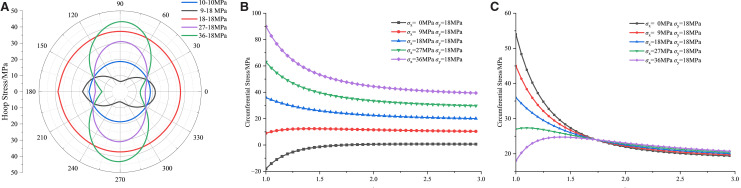
Circumferential Stress Distribution on the Blasthole Wall under Different Lateral Pressure Coefficients (a) Numerical model circumferential stress (b) Horizontal Hole Stress (Formula (4)) (c) Vertical circumferential stress (Formula (4)).

Figs 6,7 illustrate that the original rock stress field induces stress redistribution near the blast hole, leading to stress concentration. This effect intensifies with increasing stress intensity, significantly impacting rock stability and blasting efficacy. Positive values denote tensile stress, while negative values indicate compressive stress.

As the k value increases, the hoop stress concentration intensifies, creating a high-stress zone near the hole wall. For k values less than 1, compressive stress concentrates primarily on the left and right sides of the hole (θ = 0°, 180°) due to lower horizontal tectonic stress. Conversely, when k exceeds 1, the compressive stress shifts to the top and bottom (θ = 90°, 270°), indicating anisotropic stress distribution in the rock mass. At k = 1, hoop stress around the hole wall is uniformly distributed, with stress appearing similar in all directions. As the distance from the hole wall increases, hoop stress gradually decreases while radial stress rises. Beyond several pore diameters, due to stress release and redistribution in the rock mass, hoop and radial stress distributions revert to the initial stress conditions. The stress concentration coefficient at the borehole wall is approximately double that of the original rock stress field. According to elastic mechanics, the stress at a distance r from the center of the surrounding rock is given by [25]:


$$\left\{ \begin{array}{c} \sigma_{r}=\frac{\text{1}}{\text{2}}(\sigma_{x}+\sigma_{y})(1-\frac{r_{b}^{2}}{r^{2}})-\frac{\text{1}}{\text{2}}(\sigma_{x}-\sigma_{y})(1+3\frac{r_{b}^{4}}{r^{4}}-4\frac{r_{b}^{2}}{r^{2}})\cos2\beta \\ \sigma_{\theta }=\frac{\text{1}}{\text{2}}(\sigma_{x}+\sigma_{y})(1+\frac{r_{b}^{2}}{r^{2}})+\frac{\text{1}}{\text{2}}(\sigma_{x}-\sigma_{y})(1+3\frac{r_{b}^{4}}{r^{4}})\cos2\beta \\ \tau_{r\theta }=\tau_{\theta r}=\frac{\text{1}}{\text{2}}(\sigma_{x}-\sigma_{y})(1-3\frac{r_{b}^{4}}{r^{4}}+2\frac{r_{b}^{2}}{r^{2}})\sin2\beta \end{array}\right.$$ (1)

Where, *σ*_*r*_ and *σ*_*θ*_ denote the radial and circumferential stresses of the rock surrounding the hole wall due to ground stress, MPa; *τ*_*rθ*_ and *τ*_*θr*_ signify the shear stresses of the rock around the hole wall, MPa; *σ*_*x*_ and *σ*_*y*_ represent the ground stresses in the horizontal and vertical orientations, respectively, MPa; *r* indicates the distance from any point in the rock to the center of the hole, m; *r*_*b*_ is the hole’s radius, m; *β* is the angle between any direction and the axis of the gun hole, (°).

The stress distribution formula (1) derived from elasticity theory is primarily used to explain the trend of stress concentration around the borehole under different lateral pressure coefficients k. Under actual high ground stress conditions, the specific magnitudes of stress around the borehole may deviate from this elastic solution. For high-stress scenarios such as the present case, we used numerical simulation (HJC model) to obtain a stress distribution that more closely reflects reality. The simulation results (Fig 7a) showed that the orientation of stress concentration matched the trend predicted by elasticity theory, thereby confirming the applicability of the elastic analysis to this case.

To plot Equation (1) using Python, as depicted in Fig 7 (b)(c), consider how hoop stress is influenced by the lateral pressure coefficient and the distance from the hole center in both horizontal and vertical directions. In both directions, hoop stress decreases with an increase in the lateral pressure coefficient and increases with greater distance from the hole center.

In blasting engineering, the borehole location and the stress state of the surrounding rock mass are crucial for the blasting effect and the subsequent damage pattern of the rock mass. Initially, the hoop stress is greater in the vertical direction than in the horizontal. As the distance increases, the hoop stress aligns with σ_x_ horizontally and σ_y_ vertically, indicating a transition from local disturbance to global equilibrium in the stress field. Consequently, post-blasting rock mass damage predominantly aligns with the direction of the maximum principal stress. This observation aligns with the circumferential stress distribution depicted in Fig 7, confirming the analysis’s accuracy and reliability.

### 4.2. Analysis of Crack propagation by the superposition of dynamic and static loads

**Fig 8:**
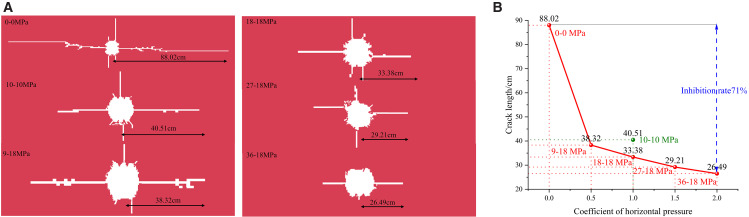
Effect of primary rock stress on crack growth in shaped charge blasting (a) Model crack propagation length (b) Crack propagation ultimate length line graphTo accurately assess crack propagation dynamics, the main crack length in case 5 (27−18 MPa) was measured every 20 μs. As shown in Fig 9(a) and based on rock blasting theory [26], the period from 0 to 60 μs represents the initial crack development phase. During this phase, the shaped charge jet exhibits high energy density and penetration capability, forming preliminary guided cracks near the blast hole wall. This jet directs subsequent crack propagation. From 60 to 130 μs, cracks aggregate and propagate, with the propagation rate increasing due to stress waves. After 130 μs, the crack enters a stabilization phase where quasi-static stress further drives propagation. By 160 μs, the crack reaches a relatively stable propagation stage, with a slowed yet ongoing propagation. Residual strain energy in the rock continues to promote crack extension and interconnection, although stress wave energy and velocity diminish. At this stage, cracks form but fail to meet the technical specifications for roof cutting and retention, which require “the opening position to be a line, the longitudinal angle a plane, and the horizontal base a line”.

Figs 8(a) and 8(b) illustrate a decreasing trend in blast-induced crack length with increasing stress levels. Specifically, measured lengths are 88.02 cm, 40.51 cm, 38.32 cm, 33.38 cm, 29.21 cm, and 25.49 cm. For instance, under Condition 6 (σ_x_ = 36 MPa, σ_y_ = 18 MPa, k = 2), the crack’s final propagation length in the cumulative energy direction (horizontal) is 25.49 cm. This represents a 71% inhibition compared to the 88.02 cm crack length under no in-situ stress (Condition 1). Additionally, cracks are notably scarce in non-cumulative directions, indicating a strong inhibitory effect of in-situ stress on crack propagation. Particularly in the horizontal cumulative direction, crack length significantly decreases with increasing in-situ stress, while vertical crack propagation is also notably restrained.

The principle of stress superposition dictates that in deep high-stress settings, stress waves from blasting interact with the existing in-situ stress, causing heightened local stress concentration in the rock mass, especially at crack tips. Elevated tensile stress at these tips increases the likelihood of tensile failure. As in-situ stress rises, the post-superposition stress concentration amplifies, facilitating crack tips reaching their tensile strength threshold. Consequently, this process expedites crack closure or impedes further propagation.

Hence, in practical construction, aligning the cumulative energy direction with the maximum principal stress direction in the roadway cross-section is crucial. This strategy leverages the concentrated energy within the cumulative jet to surmount the significant confinement along the maximum principal stress direction. Consequently, this alignment maximizes the efficiency of crack propagation while reducing the negative impacts of in-situ stress.

**Fig 9:**
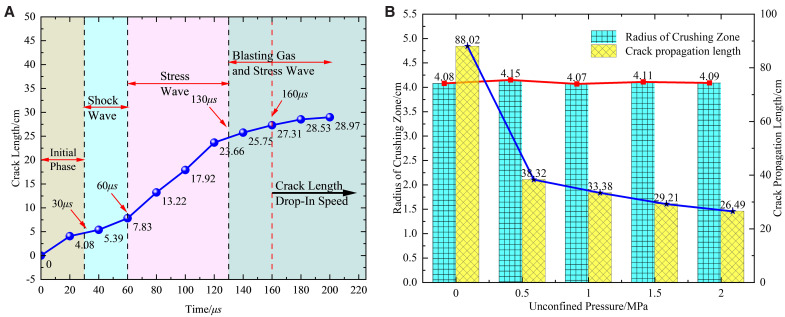
Shaped charge blasting into cracks and crack length extension (a) Shaped charge blasting process (27−18MPa) (b) Crack propagation length.

Fig 9(b) illustrates that the fragmentation zone around the blast hole remains largely unchanged as stress increases. Despite the corresponding rise in shock wave intensity, the rock material’s proximity to its failure threshold constrains the expansion of the crushing zone, preventing significant growth with further stress.

### 4.3. Analysis of fractal dimension of rock mass damage and elastic vibration energy

Post-detonation damage evolution in rock exhibits distinct fractal characteristics [27]. Consequently, the fractal box dimension is employed to quantify the damage variable of rock mass in analyzing damage from shaped charge blasting. The blasting crack damage images are binarized into black and white, and then divided into boxes with side length *δ*_*i*_. The number of boxes covering the blasting crack is denoted as *N(δ*_*i*_*).* The fractal dimension *D* is mathematically expressed as:


$$D=-\underset{l\to 0}{\lim}\frac{\log N(\delta_{i})}{\log\delta_{i}}$$ (2)

In practical calculations, the square box length δi is finite. As *δ*_*i*_ approaches zero, the ratio of *δ*i** to *N(δ*_*i*_*)* converges, allowing the data points to fit a linear equation in a double logarithmic coordinate system. The slope of this line, *D*, represents the fractal dimension.


$$\log N(\delta_{i})=-D\log(\delta_{i})+b$$ (3)

Damage fractal size D is computed first using the damage cloud map output from numerical simulation. By setting distance and grayscale threshold, the roughly ring-shaped broken area close to hole wall and the extended crack are separated in shape so that the subsequent box counting method reflects the crack complexity.

**Fig 10:**
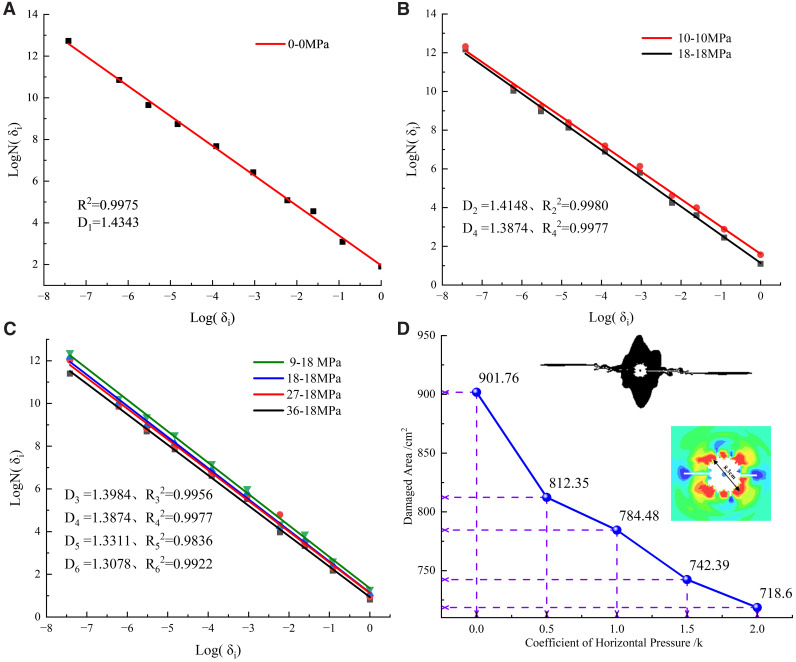
The fractal dimension fitting line of blasting crack under different side pressure coefficients (a) Unconfined pressure (b) Hydrostatic confinement pressure (c) Various lateral pressure coefficients (d) Fracture damage zone.

As shown in Fig 10, fractal dimension data of all rock blasting cracks can be fitted well by a straight line. Under the stress condition of 0 MPa, the crack formed by shaped charge blasting is not limited by the stress, and the fractal dimension (*D*_1_ = 1.4343) is the largest. When the lateral pressure coefficient *K* is 0.5, 1, 1.5 and 2.0 respectively, the fractal dimension *D* of blasting crack is 1.3984, 1.3874, 1.3311 and 1.3078 respectively, and all of them are lower than *D1* value.

The damage area is defined according to the element damage variable (*D*) output by the numerical simulation. The method is: output the damage variable (history_var) and set the NEIPH of the first TAB of DATABASE_EXTENT_BINARY to No.1, and calculate the total projected area of the model plane, which describes the area of the total spatial distribution range of blasting-damaged rock mass. Damage areas under different lateral pressure coefficients k are shown in Fig 10(d).

**Fig 11:**
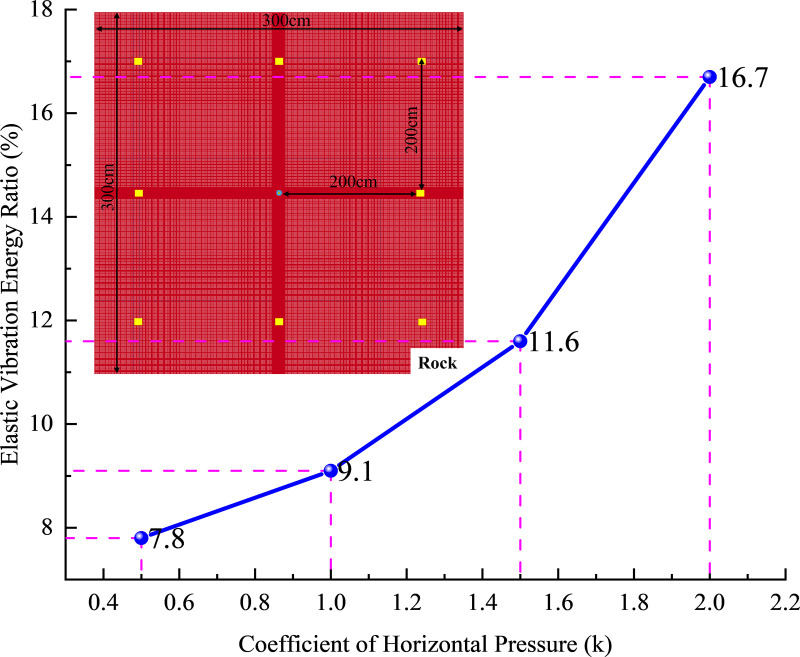
Proportion of Elastic Vibration Energy under Different In-situ Stress Conditions.

Fig 10(d) shows that as the lateral pressure coefficient K increases, the blasting damage area decreases markedly from 901.76 cm^2^ to 718.61 cm^2^. This trend indicates that higher ground stress inhibits crack propagation. The finding is consistent with the shortened main crack length (Fig 8), the reduced damage fractal dimension (Fig 10a–c), and the increased proportion of elastic vibration energy (Fig 11).

According to fractal theory, a reduction in fractal dimension indicates diminished complexity in crack propagation. At 0 MPa stress in a free state, crack propagation exhibits inherent complexity and unpredictability, resulting in a higher fractal dimension. Conversely, under lateral pressure, the crack path becomes constrained and guided, leading to a more regular and simplified morphology, and consequently, a gradual decrease in fractal dimension.

Sanchidrián et al. [28] concluded that the vibrational energy of a sphere with radius *r* is given by.


$$E_{S}=4\pi r^{2}\rho c_{L}\int_{0}^{\infty }v^{2}dt$$ (4)

Where: $c_{L}^{\text{2}}=(\lambda^{\prime }+\text{2}\mu^{\prime })/\rho$*,* $\lambda^{\prime }{,\mu }^{\prime }$ is the Lamme constant; *v* is the particle velocity vector and magnitude,$v^{\text{2}}=v_{\text{1}}^{\text{2}}+v_{\text{2}}^{\text{2}}+v_{\text{3}}^{\text{2}}$

Given the computational efficiency and the practical engineering scenario where the hole depth significantly exceeds its cross-sectional area, the model is reduced to a plane strain problem. Consequently, formula (4) is simplified to the energy integral formula (5) with a circle of radius r.


$$E_{S}=2\pi r\rho_{m}c_{L}\int_{0}^{\infty }v^{2}dt$$ (5)

**Fig 12:**
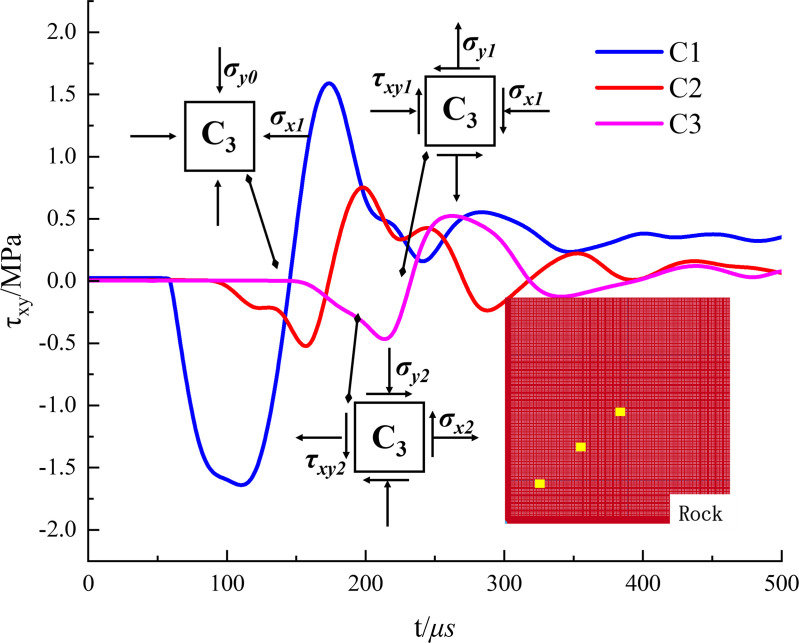
Shear stress time history curve.

To obtain far-field elastic vibration energy, eight monitoring points were established in the model, all positioned outside the crushing and crack zones created by blasting. The monitoring point systems are arranged within the model, with specific coordinates of (±200 cm, 0) and (0, ± 200 cm) on the coordinate axes, as well as (±200 cm, ± 200 cm) along the diagonals. These coordinates were then substituted into Equation (5), and the resulting calculations are presented in Fig 12.

Fig 11 illustrates that at low original rock stress levels, the elastic vibration energy ratio increases gradually with the lateral pressure coefficient. However, in high-stress plateau environments, this ratio escalates rapidly. In low-stress conditions, stress waves primarily facilitate crack expansion in the rock mass, resulting in minimal elastic vibration. Conversely, in high-stress environments, the original rock stress impedes crack expansion, allowing more blast stress wave energy to be transmitted to the rock mass, thereby inducing greater elastic vibration. When the rock mass accumulates significant elastic vibration energy, it can adversely affect the blasting area.

### 4.4. Analysis of unit stress and dynamic unloading effect of shaped charge blasting

In addition to the blast-induced stress waves and quasi-static gas pressure, the dynamic unloading effect constitutes another crucial mechanism influencing the blasting outcomes in high-stress rock masses. It refers to the radial unloading effect exerted on the surrounding rock mass after the intense fragmentation and expansion of the rock in the near-field explosion zone under high pressure. This unloading wave is capable of altering the stress path of the surrounding rock mass, thereby affecting crack propagation.

The model under condition 2 (10−10 MPa) serves as the analysis object. In the model’s first quadrant, three monitoring points, C_1_, C_2_, and C_3_, are chosen along the angle bisector (a line at a 45° angle to both the X and Y axes). These points are located 20 cm, 30 cm, and 40 cm from the model’s center, respectively, and shear stress time-history curves are plotted.

Fig 12 shows that the three-shear stress time-history curves display considerable similarity. In the illustration, *σ*_*xi*_, *σ*_*yi*_, and *τ*_*xyi*_ (i = 0,1,2) represent the values of normal and shear stress along the x and y axes at measurement point C_3_ across three distinct stages [29]. The temporal profile of shear stress at C_3_ was examined.

Initially, the measuring point unit remains in relative equilibrium with *τ*_*xy*_ at 0. Upon the arrival of the explosion stress wave, the shear stress in the rock element rapidly increases, indicating the wave’s significant impact. At 177 μs, the shear stress sign at the measuring point rotates counterclockwise due to the interaction between the explosion stress wave and the original rock stress, aligning the maximum principal stress direction more horizontally. By 330 μs, the explosion stress wave has passed, and the original rock stress once again predominates in influencing the rock unit’s stress state. The shear stress direction markedly shifts from the initial stage, now completely opposite, while the principal stress direction at the measuring point shows a clockwise rotation trend.

The accumulation of substantial elastic strain energy in rock during blasting leads to its gradual release and redistribution as the explosion stress wave propagates and attenuates. This process influences the ultimate stress state of the measurement point and the mode of crack propagation. At high stress levels, the dynamic unloading wave predominantly contributes to the internal damage zone and may exert a greater influence than the explosion stress wave [30]. During blasting, crack initiation and propagation do not necessarily align with the direction of maximum principal stress due to the in-situ stress field. Instead, they may be guided by the static stress field, ultimately bending and propagating in its direction.

The variation in shear stress direction at monitoring points reflects how the dynamic unloading wave modifies the local stress field in the rock mass. This stress rotation effect suggests that crack propagation may deviate from the original cumulative energy direction, being influenced instead by the adjusted stress field. This understanding is crucial for elucidating the occasional phenomenon of crack deviations observed during field blasting operations. The failure morphology of the rock mass is a multifaceted result of interactions among the explosion stress wave, dynamic unloading wave, release of residual elastic strain energy, and initial in-situ stress field.

## 5. Verification of on-site application effectiveness

After failure of the first test section, for easy collapse and blockage of the borehole under high stress and smooth completion of the optimization test, we checked the direction of the roadways on this working face, which was approximately parallel to the maximum horizontal principal stress direction (97°). When we are building based on this stress direction, we use a directional device to make sure charging and slotting directions of all energy-concentrated blast holes are N97°E.

**Fig 13:**
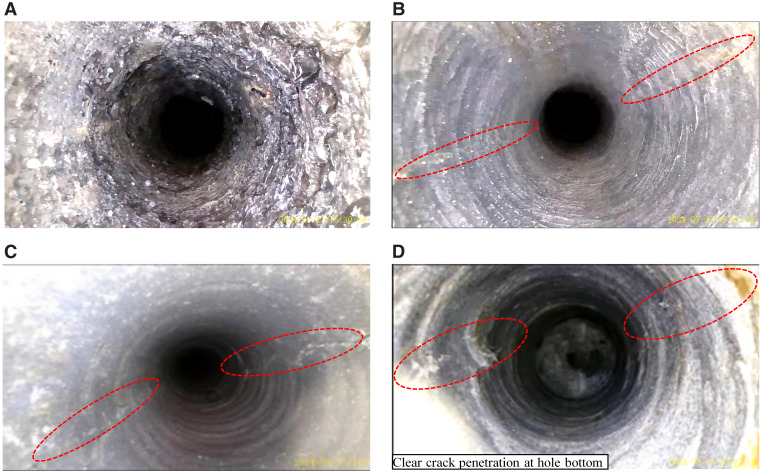
Borehole inspection map of blasting effect (a) fracture zone sandstone (b) Initial blasting crack generation (c) Formation of blasting cracks (d) Crack condition of hole bottom blasting.

During subsequent drilling operations, pipe-following drilling was used for wall protection, and the principle of “drill one hole, complete one hole, and charge one hole promptly” was strictly followed to ensure each borehole remained intact before blasting. The opening direction of the energy-concentrating pipe in the original design scheme is uniformly adjusted according to the direction of the roadway. After blasting, observation holes were built between blasting holes and a drilling camera was used to observe crack propagation. Typical peeping result is shown in Fig 13, and crack development results are shown in Fig 13. Crack development results are as follows:

Based on the key conclusion derived from the numerical simulation study in Chapter 4—“the cumulative energy direction should be aligned with the maximum principal stress direction to optimize crack propagation”—this study conducted field trial validation during subsequent construction at the 13303 working face. The opening direction of the cumulative tubes in the original design was uniformly adjusted to align with the measured maximum principal stress direction (azimuth 97°), while other blasting parameters (such as borehole spacing and charge weight) were temporarily kept unchanged to isolate and examine the effect of optimizing the cumulative energy direction. After blasting, observation holes were drilled between the blast holes to inspect crack propagation, using a borehole camera system for monitoring. Typical endoscopic results are shown in Fig 13, revealing the following characteristics of fracture development:

At a depth of 0–3 meters (shallow section), this area is situated within the plastic zone of the roadway adjacent to the rock. The predominant lithology consists of interbedded mudstone and sandstone, displaying noticeable fractured zones and stress release features. The borehole wall within the mudstone segments exhibits signs of softening and mylonitization.

At a depth of 3–6 m in the middle section, a lithological interface is identified around 4 m deep. Blast-induced fractures commence at 4.1 m depth. Subsequently, the lithology changes to sandstone with sound integrity, leading to a more uniform borehole wall. Prominent master guiding cracks are easily discernible.

At a depth of 6–8 meters (deep section), numerous continuous, interconnected directional cracks are evident. These cracks are evidently a result of the blasting from nearby boreholes. Additionally, due to the concurrent compression from substantial in-situ stress and increased abutment pressure, certain crack surfaces display minor convex deformations protruding into the observation orifice. This observation aligns with the mechanical response expected during crack propagation in high-stress settings.

Statistical analysis showed that within the charged section (approximately 5.4 m), the occurrence rate of the directional master cracks exceeded 95%, which is highly consistent with the predicted preferential crack propagation direction from the numerical simulation. Compared to the failure issues observed in the initial trials described in Section 2.3 of Chapter 2—such as “discontinuous fracture seams and severe borehole collapse”—the optimized blasting scheme demonstrated significant improvements in fracture quality, crack continuity, and directional controllability.

**Fig 14:**
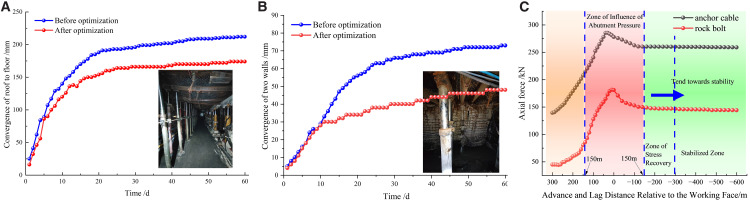
Deformation curves of roadway (a) Roof-to-floor convergence (b) Rib-to-rib convergence (c) Load Change in Anchorage Systems.

To further quantitatively validate the effectiveness, the roof-to-floor convergence and rib-to-rib convergence deformations of the roadway in the test section were monitored, as shown in Fig 14. To quantitatively assess the impact of the optimized blasting scheme on the synergistic load-bearing behavior of the surrounding rock-support system, monitoring stations were established in the test roadway section at intervals of 10 m in the advance section and 20 m in the lagging section. These stations mainly recorded roadway convergence and bolt stresses. Monitoring was conducted throughout the entire gob-side entry retention process: the presplitting blasting phase, the mining‑induced influence period during face passage, and the post‑mining stabilization period.

As illustrated in Fig 14, the zones within 150 m ahead of and behind the working face correspond to the front and rear abutment‑pressure peak zones. Under the influence of gob‑side abutment pressure, significant convergence of the roof, floor, and sidewalls, as well as pronounced roof separation, occurred in these regions. Beyond 150 m behind the face, the loads on bolts and cables tended to stabilize. At a distance of 300 m behind the face, the roadway entered a stable period.

After implementing the optimized design, the maximum roof‑to‑floor convergence during the stable period of the gob‑side entry was reduced by an average of 17.8%, and rib‑to‑rib convergence decreased by 34% compared with the non‑optimized section. These results demonstrate that the improved blast‑induced fracture quality effectively enhanced the stress distribution in the surrounding rock, thereby significantly increasing the stability of the roadside support system and the overall performance of the retained entry.

In conclusion, the field trial outcomes strongly confirm the precision of the numerical simulation findings and their practical relevance in engineering. By aligning the directional energy release with the maximum principal stress orientation, the significant hindrance posed by high in-situ stress on crack propagation was effectively mitigated. This approach notably improves the dependability and efficiency of presplitting blasting for gob-side entry retention in deep coal mines.

## 6. Conclusions

This study examined the impact of the lateral pressure coefficient on crack propagation laws during cumulative blasting for gob-side entry retention in deep coal mines using a combined methodology of numerical simulation and field experimentation. The primary findings are as follows:

(1) The stress distribution pattern around a borehole is notably influenced by the in-situ stress environment. Higher lateral pressure coefficient (k) values amplify stress concentration around the borehole, leading to a reorientation of the high-stress zone. At k = 1, circumferential stress on the borehole wall is evenly distributed, with stress concentration factor escalating proportionally with in-situ stress levels. The original stress field determines the primary direction of rock mass damage propagation following blasting.
(2) The lateral pressure coefficient (k) plays a crucial role in governing crack propagation during cumulative blasting. A higher k-value intensifies the external confinement on the rock, effectively impeding crack propagation. This is evidenced by a notable inverse relationship between the crack propagation distance in the cumulative energy direction (aligned with the maximum principal stress) and the k-value.
(3) The intricacy of blast-induced fractures and energy dispersion is governed by the in-situ stress levels. When subjected to minimal or absent in-situ stress, cracks exhibit a higher fractal dimension, intricate morphology, and a greater portion of blast energy is expended on rock fragmentation. Conversely, with an increase in the k-value, crack propagation is constrained, leading to a reduction in fractal dimension and simplification of crack morphology. Additionally, there is a notable increase in the proportion of elastic vibration energy, resulting in decreased energy efficiency.
(4) The blasting process is a multifaceted dynamic response that entails the interaction of dynamic and static loads. The stress wave generated by the explosion and the dynamic unloading effect modify the local stress distribution in the rock mass, leading to a rotation of shear stress direction in unit bodies (initially counterclockwise and then clockwise). The subsequent energy redistribution in the rock mass collectively dictates the ultimate pattern of crack propagation and stress distribution.
(5) Borehole endoscopic examinations revealed a directional crack incidence rate of up to 95% in the charged section, effectively addressing challenges like fragmented fracture seams and irregular contours inherent in the initial design. This optimization markedly improved the ultimate excavation quality and the surrounding rock stability of the gob-side entry.
